# Molecular epidemiology and clinical differentiation between *Clostridioides difficile* infection and colonization across three chicago medical centers

**DOI:** 10.1017/ash.2026.10306

**Published:** 2026-03-25

**Authors:** Andrew M. Skinner, Do Young Kim, Adam Cheknis, Michael Lin, Mary K. Hayden, Nicholas M. Moore, Amanda Harrington, Vera Tesic, Kathleen G. Beavis, Dale N. Gerding, Larry K. Kociolek, Charlesnika T. Evans, Stuart Johnson

**Affiliations:** 1 Department of Medicine and Division of Infectious Diseases, University of Utah, Salt Lake City, UT, USA; 2 Research Service, Infectious Diseases Section, VA Salt Lake City Health Care, Salt Lake City, UT, USA; 3 Department of Pathology, University of Chicago, Chicago, IL, USA; 4 Research Service, Edward Hines Jr. VA Hospital, Hines, IL, USA; 5 Department of Medicine, Division of Infectious Diseases, Rush University Medical Center, Chicago, IL, USA; 6 Department of Pathology, Rush University Medical Center, Chicago, IL, USA; 7 Department of Pathology, Loyola University Medical Center, Maywood, IL, USA; 8 Division of Pediatric Infectious Diseases, Ann & Robert H. Lurie Children’s Hospital of Chicago, Chicago, IL, USA; 9 Department of Preventive Medicine; Center for Health Services and Outcomes Research Institute for Public Health and Medicine, Northwestern University, Chicago, IL, USA; 10 Center of Innovation for Complex Chronic Healthcare, Research Service, Edward Hines Jr. VA Hospital, Hines, IL, USA; 11 Department of Medicine, Loyola University Medical Center, Maywood, IL, USA

## Abstract

In a retrospective cohort study at three Chicago academic medical centers, 22/81 (27.2%) patients with a clinically adjudicated positive *C. difficile* test result were colonized. Recent antibiotic usage predicted true infection (adjusted odds ratio: 4.38 for infection). Restriction endonuclease analysis(REA) group Y (29.3%) replaced BI (9.3%) as the dominant strain.

## Introduction

*Clostridioides difficile* infection (CDI) is the leading cause of healthcare-associated infectious diarrhea in the United States, responsible for up to 500,000 infections and 30,000 deaths annually.^1^ The economic burden approaches nearly $5 billion annually reflecting a significant healthcare cost.^2^ Consequently, the Centers for Disease Control and Prevention classified *C. difficile* as an “Urgent” threat in 2019.^3^

In the early 2000s, CDI incidence, morbidity, and mortality increased significantly.^4^ This was driven primarily by the epidemic strain which was recognized as restriction endonuclease analysis (REA) group BI, PCR Ribotype (RT) 027.^4^ Notably, a 2009 Chicago surveillance study found that BI/RT027 accounted for 61% of all infections, which were predominately hospital-onset CDI (HO-CDI).^5^ However, over the past 10 years there has been a notable decrease in HO-CDI as well as in the incidence of BI/RT027-associated CDI.^1,6^ This epidemiologic shift has seen HO-CDI rates fall while community-onset CDI (CO-CDI) rates remain constant.^1^ As BI/RT027 incidence has decreased, other strain groups such as REA group Y (RT014/020) have become more prominent.^6^

To assess these changes in molecular epidemiology, we performed a retrospective analysis of patients with a positive *C. difficile* test result at three major Chicago academic hospitals to determine the contemporary clinical and molecular epidemiology of *C. difficile*.

## Methods

This study was a retrospective, multicenter cohort study of patients with a positive *C. difficile* nucleic acid amplification test (NAAT) resulting from unformed stool collected between September 1, 2021 and October 7, 2021, at Rush University Medical Center, Loyola University Medical Center, and the University of Chicago Medical Center. The study included 81 unique patients for whom a residual stool specimen was available for REA typing. The study was approved or deemed exempt by each institution’s institutional review board.

We reviewed electronic medical records for baseline demographics, comorbidities, immunocompromised status, and relevant medication exposures. Standard CDC surveillance definitions were used for LabID surveillance events (CO-CDI, community-onset healthcare-associated CDI [COHA-CDI], and HO-CDI).^7^ To distinguish CDI from *C. difficile* colonization, two infectious diseases physicians independently reviewed cases and were blinded to REA typing during review. CDI was defined as ≥3 unformed stools over 48 hours not attributable to alternative diagnosis. Cases were further classified as primary CDI (no previous documented positive *C. difficile* test) or recurrent CDI (previous CDI within 8 wk). To address ambiguous cases such as laxative exposure or chronic noninfectious diarrhea, reviewers assessed for symptom resolution. If patients were on a laxative and laxatives were discontinued without CDI therapy, these cases were classified as *C. difficile* colonization. If patients received CDI therapy and failed to show any reduction in the stool frequency, these cases were classified as *C. difficile* colonization. Disagreements were resolved by a third infectious diseases physician.

Residual stool samples were shipped to the Edward Hines Jr., VA hospital. Samples were inoculated on TCCF agar and incubated anaerobically at 37 °C. Purified cultures underwent REA typing as previously described by Clabots *et al.* ^8^

χ^2^, Fisher’s exact test, two-sample *T* test, and Wilcoxon signed rank tests were used for unadjusted comparison between CDI and colonization, as appropriate. Variables with a *P* value < .25 were included in a multivariable logistic regression model. A stepwise backward elimination procedure based on Akaike Information Criterion was used to select the most parsimonious model.

## Results

Of the 81 unique patients included, median age was 61 years (IQR: 50–71); 55.6% (45) were female, 60.5% (49) were hospitalized, 40.7% (33) were immunocompromised, and 65.4% (53) had received systemic antibiotics within 90 days prior to their positive *C. difficile* test result. Upon physician review, 72.8% (59) were classified as CDI, while 27.2% (22) were classified as *C. difficile* colonization. CDI classifications based on standard epidemiologic surveillance criteria differed from those based on clinical assessment. Among the 42 cases classified as CO-CDI by laboratory diagnosis, 35.7% (15) were reclassified as *C. difficile* colonization.

**Figure 1.**
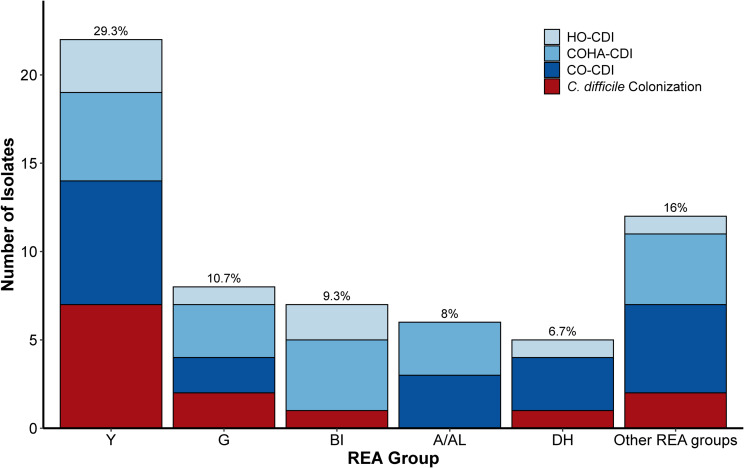
Prevalence and epidemiological status of circulating *C. difficile* REA groups at three Chicago-area Hospitals in 2021. Each bar represents the overall prevalence of the given REA group as indicated. The percentage above each bar represents the overall prevalence of that REA group among all isolates that underwent REA typing (*n* = 75). The 60 isolates presented include REA groups: Y (*n* = 22), G (*n* = 8), BI (*n* = 7), A/AL (*n* = 6), DH (*n* = 5), and other REA groups (*n* = 12). The colored segments indicate the number of isolates from patients with colonization, community-onset CDI (CO-CDI), community-onset, healthcare-associated CDI (COHA-CDI), or hospital-onset CDI (HO-CDI). Other REA groups include REA groups: BK (*n* = 2), BM (*n* = 3), CF (*n* = 2), E (*n* = 1), F (*n* = 1), J (*n* = 1), L (*n* = 2). Nonspecific REA group isolates (*n* = 15) which represented isolates infrequently encountered in our database were excluded from the Figure. For reference the major REA groups typically correlate with the following PCR Ribotypes (RT): REA group Y (014/021); G (002); BI (027); A/AL (054/056); DH (106); BK (078/126); BM (103); CF (017); E (005); F (046); J (001); L (003). [Kociolek LK, *et al*. Anaerobe 2018;54:1–7].

*C. difficile* was recovered from 75 of 81 specimens. Of the 75 isolates that were available for REA typing, REA group Y was most common (29.3%; 22), followed by REA group G (10.7%; 8), and REA group BI (9.3%; 7) (Figure 1). Notably, 31.8% (7/22) of the REA group Y isolates were from colonized patients. In contrast, only one of the seven (14.3%) REA group BI isolates were from a colonized patient.

Multivariable logistic regression revealed that patients with recent systemic antibiotic exposure had increased odds of being classified as CDI by 4.38 times (adjusted odds ratio [aOR] 4.38; 95% CI: 1.49–13.71) (Table 1). There was a nonsignificant trend towards laxative use decreasing the odds of being classified as a CDI (aOR: 0.24; 95% CI: 0.04–1.30), suggesting that laxative-associated diarrhea may act as a diagnostic confounder.

**Table 1.** Baseline demographics and clinical characteristics, unadjusted and multivariable analysis of patients with a positive *Clostridioides difficile* NAAT

Characteristic	*N* = 81			
Female (%)	45 (55.6)			
Median age, years (IQR)	61 (50–71)			
Age ≥65 (%)	36 (44.4)			
Hospitalized patient (%)	49 (60.5)			
Charlson Comorbidity Index (IQR)	4 (1–6)			
†Immunocompromised (%)	33 (40.7)			
PPI/H2RA use (%)	31 (38.3)			
††Laxative use (%)	8 (9.9)			
‡Recent systemic antibiotic use (%)	53 (65.4)			
Positive CD test in past 3 months (%)	26 (32.1)			
Hospital				
Hospital 1 (%)	49 (60.5)			
Hospital 2 (%)	12 (14.8)			
Hospital 3 (%)	20 (24.7)			
‡‡Diarrhea (%)	71 (87.7)			
Abdominal pain (%)	27 (33.3)			
Temperature, Celsius (IQR)	36.8 (36.5–37.1)			
Median WBC (IQR)	9.3 (5.9–12.3)			
Median creatinine (IQR)	.98 (.70–1.61)			
LabID surveillance events				
CO-CDI (%)	42 (51.9)			
COHA-CDI (%)	26 (32.1)			
HO-CDI (%)	13 (16.0)			
Clinical epidemiology by physician review				
CO-CDI (%)	27 (33.3)			
COHA-CDI (%)	23 (28.4)			
HO-CDI (%)	9 (11.1)			
Colonization (%)	22 (27.2)			

Unadjusted analysis of factors associated with *C. difficile* infection (CDI) versus colonization				
Characteristic	CDI (*n* = 59)	Colonization (*n* = 22)		*p*-value
Female (%)	33 (55.9%)	12 (54.5%)		1.00
Age (IQR)	62 (51–71)	58 (43–79)		.64
Age ≥65 (%)	27 (45.8%)	9 (40.9%)		.80
Inpatient (%)	32 (54.2%)	17 (77.3%)		.08
Charlson Comorbidity Index (IQR)	4 (1–5)	4 (1–7)		.97
†Immunocompromised (%)	25 (42.4%)	8 (36.4%)		.80
PPI/H2RA use (%)	20 (33.9%)	11 (50.0%)		.21
††Laxative use (%)	3 (5.1%)	5 (22.7%)		.03
‡Recent systemic antibiotics (%)	44 (74.6%)	9 (40.9%)		.01
Positive CD test in past 3 months (%)	20 (33.9%)	6 (27.3%)		.79
Temperature, Celsius (IQR)	36.7 (36.6–37.1)	36.8 (36.4–37.1)		.63
Median WBC (IQR)	9.6 (5.5–13.1)	7.9 (6.4–10.9)		.74
Creatinine (IQR)	1.07 (0.68–1.64)	0.83 (0.70–1.22)		.85

Multivariable logistic regression of predictors of *C. difficile* infection				
Characteristic	cOR (95% CI)		aOR (95% CI)	
‡Recent systemic antibiotic exposure	4.24 (1.53–12.29)		4.38 (1.49–13.71)	
††Laxative exposure	0.18 (0.03–0.82)		0.24 (0.04–1.30)	
PPI/H2RA exposure (%)	0.51 (0.19–1.39)		–	
Inpatient diagnosis	0.35 (0.10–1.01)		0.37 (0.10–1.18)	

IQR, Interquartile range. PPI, Proton pump inhibitor; H2RA, Histamine 2 receptor antagonist; WBC, White blood cell count; CDI: *C. difficile* infection; CO-CDI: Community-onset CDI; COHA-CDI: Community-onset, healthcare-associated CDI; HO-CDI: Hospital-onset CDI; cOR: Crude odds ratio; aOR: adjusted odds ratio; CI.

Laboratory (WBC, creatinine) data collected on day of positive test.

†Immunocompromised: HIV with a CD4 < 200 and not on HIV therapy, autoimmune disease, active solid organ malignancy, lymphoma, leukemia, multiple myeloma, receipt of medication with known immunosuppressing effects such as long-term steroids (eg, 20 mg of prednisone or equivalent for ≥20 d).

††Laxative Use: Laxative use within 48 hours of positive *C. difficile* test.

‡Recent systemic antibiotics: Antibiotics received within 90 days of positive *C. difficile* test.

‡‡Diarrhea: 3+ unformed stools.

## Discussion

This multicenter surveillance study demonstrates a change in the clinical and molecular epidemiology of *C. difficile* in the city of Chicago. The epidemic BI/RT027 strain, which accounted for 61% of isolates in 2009 as reported by Black *et al* has been replaced by the more endemic REA group Y (29.3%) as the most prevalent strain.^5^ This shift corresponds with a dramatic reduction in HO-CDI (57% in 2009 vs 11.1% in our 2021 cohort) and a proportional rise in CO-CDI.^5^

The changing epidemiology is possibly attributable to interventions, primarily antimicrobial stewardship and infection control measures, which targeted HO-CDI and consequently impacted the incidence of CDI caused by BI/RT027, which is fluoroquinolone-resistant.^9^ These data indicate that as the molecular epidemiology has shifted from strains associated with acute HO-CDI towards those linked with colonization, our understanding of *C. difficile* pathogenesis may change. Previous CDI models relied on data that demonstrated CDI developed as the result of new acquisition of *C. difficile* and rapid symptom development.^10^ Our findings support a model where infections may represent a transition from established colonization to CDI following microbiome disruptions. These data reveal that antibiotics remained a strong predictor of infection, and nearly one-third of REA group Y isolates were found in colonized patients, suggesting a reservoir that could readily transition to infection with antibiotic exposure.

Our study also highlights the challenges of NAAT-only diagnostics, as more than 27% of positive tests were deemed as colonization upon chart review. A reliance on NAATs may inflate CDI rates and complicates epidemiologic surveillance. Notably, our colonization rate was determined by a rigorous adjudication process designed to assess for common confounders, such as laxative use. Future studies should consider incorporating both objective measures and clinical assessments to differentiate infection from colonization to accurately track circulating pathogenic strains.

The study’s strengths include its multicenter design and physician-led adjudication of infection status. The primary limitation is the small sample size (*n* = 81) gathered over a five-week period at three academic hospitals. This significantly limits the power of the multivariable analysis and the generalizability of strain prevalence. Lastly, six stool specimens were culture-negative and we could not confirm the REA typing among these patients (colonization = 3, CO-CDI = 1, and COHA-CDI = 2).

Our study documents a shift in the *C. difficile* epidemiology in Chicago, with REA group Y displacing REA group BI as the dominant strain. This likely reflects a move away from healthcare-associated transmission and towards a model of community-based colonization, though these findings require validation in larger, year-round surveillance studies to identify new CO-CDI reservoirs.

## Data Availability

Data are available on reasonable requests from the corresponding author with authorization from an Institutional Review Board.
